# Distal Renal Tubular Acidosis With Sensorineural Deafness in a Saudi Female: A Case Report of an ATP6V1B1 Mutation in a Consanguineous Family

**DOI:** 10.7759/cureus.98596

**Published:** 2025-12-06

**Authors:** Abeer Alrasheed, Nouf Alyabis, Soud A Alrasheed, Reem Alrasheed

**Affiliations:** 1 Medicine, Alfaisal University College of Medicine, Riyadh, SAU; 2 Pediatric Nephrology, King Abdulaziz Medical City, Riyadh, SAU; 3 Pharmacy, King Abdulaziz Medical City, Riyadh, SAU

**Keywords:** atp6v1b1 gene mutation, consanguinity, nephrocalcinosis, renal tubular acidosis, sensorineural hearing loss

## Abstract

Distal renal tubular acidosis (dRTA) with sensorineural deafness is a rare entity inherited in an autosomal recessive manner caused by mutations in the *ATP6V1B1* gene leading to defective acidification function in the distal nephron, cochlea, and endolymphatic sac. We report the case of an 11-year-old Saudi girl with dRTA and congenital sensorineural hearing loss. Genetic testing revealed a homozygous mutation in the *ATP6V1B1* gene (c.1037C>G; p.P346R). Both parents were heterozygous carriers. This case highlights the clinical and genetic features of dRTA in a consanguineous family and underscores the importance of early genetic diagnosis and multidisciplinary management.

## Introduction

Distal renal tubular acidosis (dRTA) is a rare inherited disorder caused by the inability of the kidney to secrete hydrogen ions in the distal tubule. This causes metabolic acidosis with a normal anion gap [1]. In some patients with dRTA, there is sensorineural hearing loss [2]. The mode of inheritance is autosomal recessive. It is most frequently caused by mutations in the *ATP6V1B1* or *ATP6V0A4* genes. Mutations in the *SLC4A1* gene cause a milder form of dRTA that follows an autosomal dominant inheritance and often presents in adulthood; therefore, few pediatric cases have been reported. The *ATP6V1B1* gene encodes a subunit of the H+-ATPase pump [3]. The pump plays a role in both renal acidification and inner ear function [4]. Mutations in the gene disrupt both these processes and lead to renal and auditory manifestations [3]. The disorder is rare worldwide [1]. It is, however, more common in populations with a high rate of consanguineous marriages [5]. Consanguinity is culturally accepted and common in Saudi Arabia [5,6]. There have been limited reports from Saudi Arabia of this combined presentation. Early diagnosis and treatment are required to reduce complications. A genetic diagnosis can confirm a definitive diagnosis and direct family counseling [7,8]. We report an 11-year-old Saudi girl with dRTA and severe sensorineural hearing loss, born to first-degree cousins. Two of her older brothers were also affected. A homozygous *ATP6V1B1* mutation was identified in the patient and her affected siblings, while both parents were found to be heterozygous carriers.

## Case presentation

An 11-year-old girl was referred for evaluation of failure to thrive and hypokalemia. She was the sixth and youngest child born to a first-degree consanguineous couple. Two of her older brothers were known to have renal tubular acidosis (RTA) and deafness. She was born at 39 weeks of gestation, with a birth weight of 3.1 kg. She was found to have a hearing deficit since birth, which was confirmed using automated auditory brainstem response (AABR) testing.

On physical examination, she weighed 20.4 kg and measured 115 cm in height, both below the third percentile. She had mild bilateral genu valgum and profound hearing loss. The rest of the examination was normal. Investigations showed the following: laboratory results are summarized in Table 1. There was no pyuria, glycosuria, or hematuria. The urine anion gap was positive.

**Table 1:** Laboratory findings on presentation TCO₂: Total carbon dioxide.

Parameters	Result	Reference Range	Units
Serum sodium	136	136-145	mmol/L
Serum potassium	3.0	3.5-5.1	mmol/L
Serum chloride	114	98-107	mmol/L
TCO₂	11	22-29	mmol/L
Serum creatinine	38	53-78	µmol/L
Blood urea nitrogen	5.4	3.0-7.5	mmol/L
Serum calcium	2.4	2.1-2.55	mmol/L
Serum phosphorus	1.43	0.81-1.45	mmol/L
Serum albumin	42	35-50	g/L
Serum anion gap	14	8-16	mmol/L
Random urine sodium	50	>20	mmol/L
Random urine potassium	30	20-100	mmol/L
Random urine random urine	40	30-260	mmol/L
Urine pH	8.5	5.0-7.0	—

**Figure 1:**
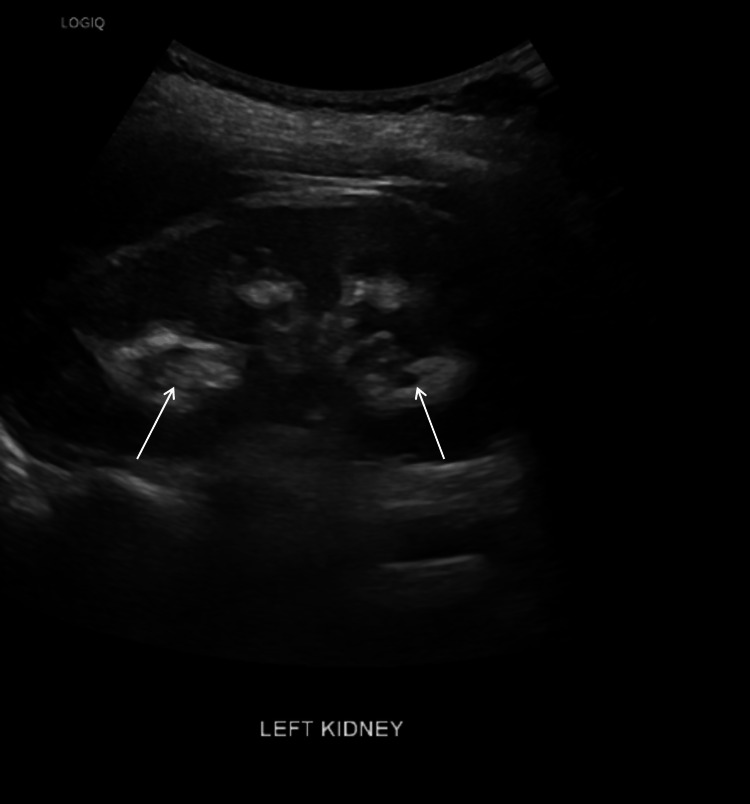
Renal ultrasound of the left kidney demonstrating hyperechoic medullary pyramids (arrows), consistent with medullary nephrocalcinosis

Renal ultrasound (Figure 1) revealed bilateral medullary nephrocalcinosis. Audiometry confirmed severe bilateral sensorineural hearing loss.

These findings confirm the diagnosis of distal RTA (dRTA) with sensorineural deafness. After obtaining consent from parents, genetic analysis was performed that showed a homozygous pathogenic variant in the *ATP6V1B1* gene c.1037C>G; p.P346R.

Subsequently, genetic screening for the family members was carried out after obtaining consent. The results showed that both parents and one brother were heterozygous for the mutation, while the two brothers with RTA and deafness had the homozygous *ATP6V1B1* gene mutation (c.1037C>G; p.P346R). The remaining two brothers were not affected.

**Figure 2:**
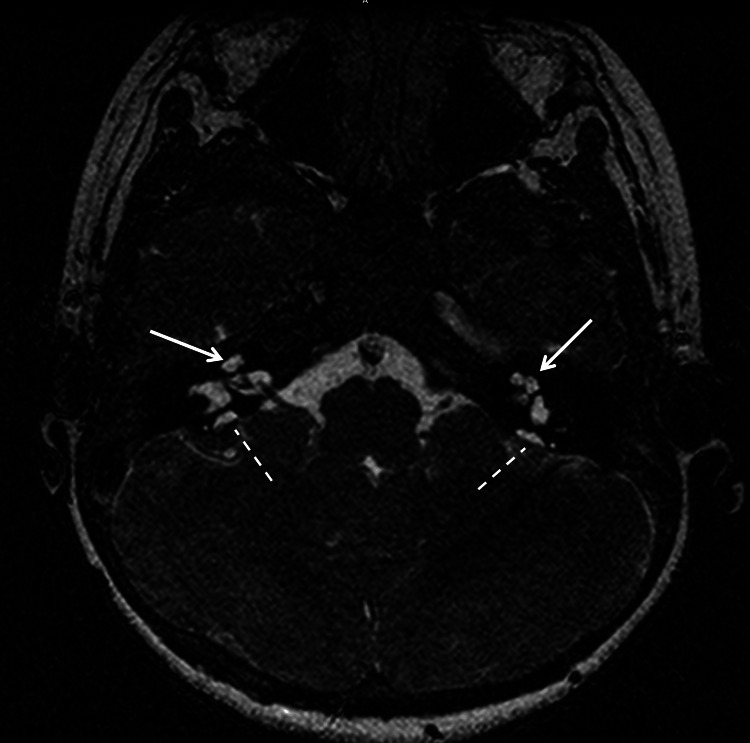
MRI of the brain showing bilateral malformed cochleae (solid arrows) and endolymphatic sacs (dotted lines), both representing Mondini’s malformation

Brain MRI revealed bilateral cochlear malformation consistent with Mondini's deformity and dilated vestibular aqueducts (right: 1.9 mm; left: 2.4 mm) (Figure 2). These structures communicated with small extra-axial fluid signals posterior to the mastoid bones, representing endolymphatic sacs. The vestibule and lateral semicircular canals were well developed and normally configured. The above-described findings were consistent with bilateral Mondini’s malformation and bilateral endolymphatic sacs.

She was treated with Polycitra-K (citric acid/K-citrate/Na citrate) to provide 6 mmol/kg/day of bicarbonate equivalent. Aural rehabilitation with hearing aids and speech therapy was initiated, and she was referred to an otolaryngologist for consideration of a cochlear implant to support her language development.

## Discussion

Acid-base homeostasis is critical for normal cellular function, growth, and development. The kidneys play an important role by reabsorbing bicarbonate, mainly in the proximal tubules, and by secreting acid in the distal tubules and collecting ducts of the nephrons. The net acid secretion is mediated largely by an energy-dependent proton pump (H+-ATPase) located in the apical membrane of α-intercalated cells of the distal nephron [9,10]. Failure of the urine acidification process results in dRTA.

In families with dRTA and early-onset hearing loss, the putative mutation was identified in the *ATP6V1B1* gene encoding the B subunit of the H+-ATPase pump located in the apical surface of the α-intercalated cells in the distal tubule and is also expressed in the human cochlea and endolymphatic sac epithelium. An active acidification process constantly occurs in the ear to maintain endolymph pH near 7.4 in the cochlea and closer to 6.6 in the endolymphatic sac. Failure of this process leads to sensorineural deafness [11]. The mutation identified in our patient and her older brothers explains the occurrence of dRTA and early-onset deafness. Mutations in *ATP6V1B1* were first identified as a cause of dRTA and deafness by Karet et al. in 1999 [6]. Since then, several variants of these gene mutations have been identified in affected individuals from various populations worldwide, including Saudi Arabia [12]. Most reports indicate its occurrence in consanguineous families and describe delayed diagnosis because of overlapping symptoms with more common pediatric conditions such as rickets and dehydration [7]. Consanguineous marriage is socially acceptable in Saudi Arabia [8]. The total consanguinity rate is approximately 57.7%, leading to a higher prevalence of autosomal recessive diseases like dRTA and deafness [8]. The current report of three siblings and *ATP6V1B1* mutations illustrates this risk.

Gene testing based on sequencing of exon 12 at the *ATP6V1B1* gene for the whole family members of the affected individual helps in the diagnosis and identification of carriers and forms the basis for counseling and clinical management [13]. The main treatment of dRTA is alkali therapy to correct metabolic acidosis and prevent some complications such as growth failure, kidney stones, and renal failure [3,14]. Alkali therapy does not alter the course of the sensorineural deafness. Management of such cases is complex and requires long-term follow-up by a multidisciplinary team, consisting of a pediatric nephrologist, otolaryngologist, urologist, genetic counselor, and speech therapist [15].

## Conclusions

This case report describes three siblings from a Saudi consanguineous family with dRTA, sensorineural deafness, and *ATP6V1B1* gene mutation. Imaging confirmed Mondini's malformation and dilated vestibular aqueducts. Early diagnosis and genetic confirmation are essential for appropriate management and family counseling. A multidisciplinary care plan is necessary to address both renal and auditory complications of this rare condition.
